# Maximum likelihood estimation under the Emax model: existence, geometry and efficiency

**DOI:** 10.1007/s00362-025-01673-2

**Published:** 2025-06-10

**Authors:** Giacomo Aletti, Nancy Flournoy, Caterina May, Chiara Tommasi

**Affiliations:** 1 ADAMSS Center, Università degli Studi di Milano, V. Saldini 50, 20133 Milan, Italy; 2 University of Missouri, 600 S. State St., Apt 408, Bellingham, WA 98225 USA; 3 Dipartimento DiSEI, Università del Piemonte Orientale, V. Ettore Perrone 18, 28100 Novara, Italy; 4 Department of Mathematics, King’s College London, Strand Campus, London, WC2R 2LS UK; 5 DEMMQ, Università degli Studi di Milano, V. Conservatorio 7, 20122 Milan, Italy

**Keywords:** D-optimum experimental design, Dose-finding, Nonlinear regression, Score modification

## Abstract

This study focuses on the estimation of the Emax dose–response model, a widely utilized framework in clinical trials, experiments in pharmacology, agriculture, environmental science, and more. Existing challenges in obtaining maximum likelihood estimates (MLE) for model parameters are often ascribed to computational issues but, in reality, stem from the absence of a MLE. Our contribution provides new understanding and control of all the experimental situations that practitioners might face, guiding them in the estimation process. We derive the exact MLE for a three-point experimental design and identify the two scenarios where the MLE fails to exist. To address these challenges, we propose utilizing Firth’s modified score, which we express analytically as a function of the experimental design. Through a simulation study, we demonstrate that the Firth modification yields a finite estimate in one of the problematic scenarios. For the remaining case, we introduce a design-augmentation strategy akin to a hypothesis test.

## Introduction

The Emax model provides a useful dose–response model for wide variety of applications. It is a form of the Hill and the Langmuir equations. Archibald Vivian Hill introduced mathematics and quantitative thinking to physiology with his hypergeometric model of ligands binding to macromolecules as a function of the ligand concentration (in particular, oxygen binding to hemoglobin, and calcium ions binding to a protein in muscle contraction Hill 1909, 1910a, b; Barcroft and Hill 1910), and this led to his subsequent Nobel Prize. In their first mathematical formulation of drug kinetics (Barcroft and Hill 1910), Hill’s equation is re-written (by taking log concentration) as a logistic function [with its characteristic sigmoid shape ranging between a known constant lower asymptote (often zero) and an unknown upper asymptote]; it includes the later well-known model of Michaelis and Menten (1913) as a special case. The Emax model was derived again a few years later (Langmuir 1916) starting from a mathematical description of the adsorption of species onto simple surfaces.

Now the Emax model enjoys standard use in a variety of fields including physiology, pharmacology, clinical trials, biochemistry, agriculture and environmental science (e.g., Bretz et al. 2010; Baker 2010; Baker et al. 2016; Han et al. 2011; Holford and Sheiner 1981; MacDougall 2006; Denney et al. 2017; Rath et al. 2022). The 4-parameter logistic function is an extension of the 3-parameter logistic that includes an unknown lower asymptote; it is used when the assumption of a known lower asymptote in the logistic formulation is unrealistic.

In this study, we focus on the three-parameter version, which is frequently used in dose–response studies where it is often assumed that the mean response can be described by a simple concave function that increases monotonically with a covariate $x\in \mathcal {X}$, such as dose or stress (see Leonov and Miller 2009; Chen et al. 2023). Other aspects of this model have been studied by Dette et al. (2010), Dragalin et al. (2010), and Flournoy et al. (2021).

Although the maximum likelihood estimates (MLEs) of the model parameters are asymptotically consistent, it is well-known that uncertainty in finite samples may lead to the non-existence of the MLE. There also is a large literature on other convergence problems associated with Emax parameters estimation algorithms; see, for example, Fedorov and Leonov (2013), Flournoy et al. (2020) and Chen et al. (2023). We analyze the same convergence problems theoretically (not from a numerical point of view), exposing geometric features important to the application of likelihood methods under the Emax model.

Our main concern is to guarantee (as much as possible) a finite estimate of the model parameters. It is well known that the D-optimal design (which has three support points) leads to the most precise parameter estimate. Herein, we show that (among all the three-point designs) the D-optimal design has a low probability that the MLE does not exist, which is a very useful property. For analytical and geometric tractability, we focus only on three-point experimental designs. Three-point designs are common choice in experimental practice; in addition, they lay the foundation for extensions to more complex designs. Provided that observed means at the design’s support points have an increasing concave shape, we are able to give an analytic expression of the MLE vector as a function of the data. We prove that if the observations do not satisfy this geometric shape, then the MLE does not exist. In particular, we identify two different scenarios (with several sub-cases) where the MLE fails. We call these problematic scenarios as “Case 1” and “Case 2” and provide the probabilities of observing them.

To obtain finite estimates in these unlucky cases where MLEs do not exist, we recommend estimating parameters with the roots of the modified score equations (see Firth 1993). Briefly, Firth developed a general method to reduce the bias of the MLE by modifying the score function. The roots of Firth’s modified score equations result in first-order unbiased estimators, herein called *Firth-modified estimators* (see also Kosmidis and Firth 2009a, b). But, more importantly for Emax model estimation, Firth (1993, Sect. 3.3) shows that his estimators may circumvent the problem of the non-existence MLEs even with moderate sample sizes.

To apply Firth’s method, we derive an analytical expression of Firth’s correction for the score function of the Emax model as a function of any given design. We find that Firth’s modified score leads to a finite estimate only in Case 2. Unfortunately, in Case 1 Firth’s method fails. In Case 1, however, a geometric argumentation leads to our proposal of design augmentation, which consists of identifying the region where an additional experimental point can be added to obtain a finite estimate. Let us note that this design-augmentation is proved to be equivalent to a hypothesis test on the most critical parameter of the Emax model.

In Sect. 2, we give the notation and common parameterization of the Emax model. Section 3 provides the analytic expression of the MLE with conditions for its existence and descriptions of the bad scenarios for which the MLE does not exist. In Sect. 4, we provide the explicit formula for Firth’s adjustment of the Emax score function. In Sect. 5 we face the bad scenarios, showing when Firth method succeeds as well as when a design-augmentation strategy is necessary to control the problem. Finally Sect. 6, which concludes the paper, provides practical guidelines for an experimenter resulting from our theoretical results.

## Model and notation

The Emax model (with Gaussian errors) is $y=\eta (x,\varvec{\theta })+\varepsilon$, where *y* denotes a response at dose $x\in \mathcal {X}=[a,b]$; $a\ge 0$ and $b\ge a$ are the lowest and the highest admissible doses; $\varepsilon$ is a Gaussian random error; and $\varvec{\theta }=(\theta _0, \theta _1,\theta _2)^T$ is a vector of unknown parameters that belongs to a parameter space that makes the mean response

1 $$\begin{aligned} \eta (x,\varvec{\theta })=\theta _0+\theta _1\,\frac{x}{x+\theta _2} \end{aligned}$$

an increasing and concave curve (see Sect. 2.1).

An experimental design is a finite discrete probability distribution over $\mathcal {X}$:

2 $$\begin{aligned} \xi =\left\{ \begin{array}{ccc} x_1 & \cdots & x_M \\ \omega _1 & \cdots & \omega _M \end{array} \right\} , \end{aligned}$$

where $x_i$ denotes the *i*th experimental point, or treatment dose, that may be used in the study and $\omega _i$ is the proportion of experimental units to be taken at that point; $\omega _i\ge 0$ for $i=1,\ldots ,M$ with $\sum _{i=1}^{M}\omega _i=1$ and *M* finite.

Assuming that at the dose $x_i$ we observe $n_i=\omega _i \cdot \,n$ independent responses, $y_{i1},\ldots ,y_{in_i}$ (for $i=1,\ldots ,M$), the most common estimate of $\varvec{\theta }$ is the maximum likelihood estimator (MLE). It is well known that, to improve the precision of the MLE, we should apply a D-optimal design, since it minimizes the generalized asymptotic variance of the MLE for $\varvec{\theta }$. General references on optimal design of experiments include Fedorov (1972), Atkinson et al. (2007) and Silvey (2013), while Fedorov and Leonov (2013) is specific to optimal designs for nonlinear response models. In particular, Fedorov and Leonov describe strategies for implementing D-optimal designs when they are functions of the unknown parameters. We recommend the use of sequential adaptations for the Emax model in Sect. 6.

Let

$$\begin{aligned} I(\xi ;\varvec{\theta })=\int _\mathcal {X} \nabla \eta (x,\varvec{\theta }) \nabla \eta (x,\varvec{\theta })^T d\xi (x) \end{aligned}$$

denote the Fisher information matrix of an experiment with design $\xi$ under model (1), where $\nabla \eta (x,\varvec{\theta })$ denotes the gradient of the mean response $\eta (x,\varvec{\theta })$ with respect to $\varvec{\theta }$. The D-optimal design for the Emax model (provided by Dette et al. 2010) is

3 $$\begin{aligned} \xi ^*(\varvec{\theta })= \arg \max _{\xi \in {\Xi }}\ \textrm{Det}[{I}(\xi ,;\varvec{\theta })] =\left\{ \begin{matrix} a & x^*(\theta _2) & b \\ 1/3 & 1/3 & 1/3 \end{matrix} \right\} , \end{aligned}$$

where $\Xi$ is the set of all possible designs and

4 $$\begin{aligned} x^*(\theta _2)=\frac{b(a+\theta _2)+a(b+\theta _2)}{(a+\theta _2)+(b+\theta _2)}. \end{aligned}$$

Design $\xi ^*({\varvec{\theta }})$ is said to be locally optimal because it depends on the unknown parameter value ${\varvec{\theta }}$, due to the non-linearity of $\eta (x,\varvec{\theta })$. See Sect. 6 for implementation recommendations.

### Parameter space with Interpretation

**Fig. 1:**
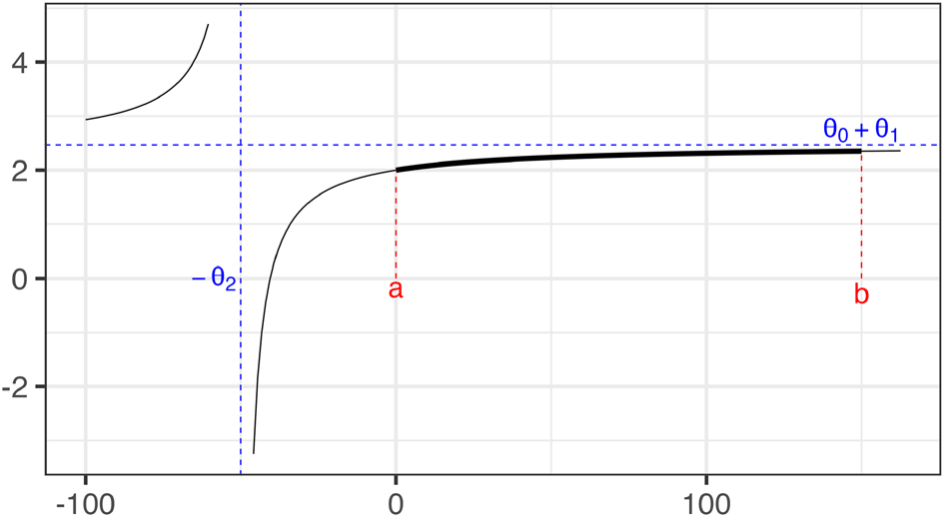
Emax mean response curve as part of a branch of a hyperbola. Black line: hyperbola with equation $\eta (x) = \theta _0 + \theta _1x /(x+\theta _2) = 2 + 0.467 x /( x + 50)$. Blue dashed lines: vertical ($x=-\theta _2$) and horizontal ($y= \theta _0+\theta _1$) asymptotes of the hyperbola. The thick part of the graph corresponds to the Emax mean response curve, which is given on the support $\mathcal {X} = [a,b]$. (Color figure online)

It is important to note for what will be discussed in this section that the equation $y=\eta (x,\varvec{\theta })$ given by model (1) on the Cartesian plane is an hyperbola with the upper horizontal asymptote $y=\theta _0+\theta _1$ and the vertical asymptote $x=-\theta _2$. The Emax mean response curve is the concave branch of this hyperbola. An example (discussed further in Sect. 3) is shown in Fig. 1.

It is often natural to assume that the dose $x=0$ is the lowest dose admissible, that is, the lower limit of the experimental space is $a=0$. In the Emax model, if $a=0$, then $\theta _0$ represents the response at dose $x=0$ (placebo effect), $\theta _1> 0$ is the asymptotic maximum effect attributable to the drug (for an infinite dose) and $\theta _2>0$ is the dose which produces the half of the maximum effect. By considering this parameter space ($\theta _1> 0$, $\theta _2> 0$) for $x \in [0,b]$, all the concave branches of the hyperbola can be fitted by the model.

To solve the computational problems and to ensure the existence of the MLE, several authors have bounded the space of the non-linear parameter $\theta _2$ in a positive compact set; for instance, Dette et al. (2012) consider $\theta _2 \in [0.015; 1500]$. However, this approach excludes some parameter values that could produce a response curve which is more closely aligned with the observed data. In this paper we solve all the computational problems by providing an analytic solution of the MLE for $\theta _2>0$, together with the exact conditions of its existence (see Sect. 3).

When the lowest dose admissible is $a> 0$, the parameter interpretation given above fails. In addition, if $a>0$ and $\theta _2 > 0$, all admissible response curves with vertical asymptote in (0, *a*) are excluded. These curves may better approximate the observed data, and hence the parametric domain of $\theta _2$ should be extended to ensure the best fit. This is motivated in detail in Sect. 2.2 by introducing a suitable reparametrization of the model. The conditions of existence and the analytic solution for the MLE are provided in Sect. 3, including for this case.

### Reparametrization when the lower boundary of admissible doses is $a>0$

In some situations, due to ethical concerns, patient exposure to placebo is not feasible, and then the mean response curve $E(Y \vert X= x)$ is considered in a domain $\mathcal {X}=[a,b]$, $a>0$. Then Model (1) can be rewritten as

5 $$\begin{aligned} E(Y \vert {X}= x) = \eta (x-a,\tilde{\varvec{\theta }})=\tilde{\theta }_0+\tilde{\theta }_1\,\frac{(x-a)}{(x-a)+\tilde{\theta }_2}, \end{aligned}$$

where

6 $$\begin{aligned} \tilde{\theta }_0 = {\theta }_0 +a \frac{{\theta }_1}{{\theta }_2 + a}, \quad \tilde{\theta }_1 = \theta _1 - a \frac{\theta _1}{{\theta }_2 + a}, \quad \tilde{\theta }_2 = \theta _2 + a. \end{aligned}$$

Model (5) represents an Emax model in a new parametrization, with a change in the coordinate to $\tilde{x} = x-a$ (this same parametrization has been considered by Chen et al. 2023). It is reasonable to expect an experimenter to be interested in estimating the new parameters since $\tilde{\theta }_0$ is the mean response for the lower dose $x=a$; $\tilde{\theta }_1$ is the asymptotic maximum effect attributable to the drug with respect to the response at the minimal dose; and $\tilde{\theta }_2$ is the dose added to *a* which produces the half of the maximum effect. Like in the case $a=0$, the constraints $\tilde{\theta }_1>0$, $\tilde{\theta }_2>0$ identify all the increasing and concave branches of hyperbola.

Next Proposition 1 shows that the Emax mean response curves are not a compact set; roughly speaking, some response curves obtained as limit of curves in model (1) do not belong to the same model. As a consequence, there are situations in which the data are such that it is not possible to find an “Emax” mean response curve that maximizes the likelihood. Instead the likelihood is maximized by one of the three limiting cases listed in Proposition 1. Consider now $\mathcal {X}=[a,b]$, $a\ge 0$, and the reparametrization (5) (which coincides with the standard parametrization when $a=0$).

#### Proposition 1

The set of increasing and concave branches of hyperbola on $\mathcal {X}$ is not locally compact in the set of bounded functions on $\mathcal {X}$ with point-wise convergence. In fact, the limit class contains other bounded functions on $\mathcal {X},$ which precisely are

1. the strictly increasing straight lines $\tilde{y} = m\,\tilde{x}+q,$ as limits of $\eta (\tilde{x},\tilde{\varvec{\theta }})$ that occurs when, for instance, $\tilde{\theta }_{0} = q,$ $\tilde{\theta }_{1} = m\tilde{\theta }_{2}$ and $\tilde{\theta }_{2} \rightarrow \infty ;$
2. the horizontal lines $\tilde{y} = q,$ that occurs when $\tilde{\theta }_{0} = q$ and (*a)* $\tilde{\theta }_{1}=0$ and any value of $\tilde{\theta }_{2}$ or (*b)* whenever $\tilde{\theta }_{1}= o(\tilde{\theta }_{2});$
3. the horizontal line $\tilde{y} = q+ q^*\mathbb {1}_{\tilde{x}>0}$ which is discontinuous at $\tilde{x}=0,$ and occurs when $\tilde{\theta }_{0} = q,$ $\tilde{\theta }_{1} = q^*\tilde{\theta }_{2},$ and $\tilde{\theta }_{2} \rightarrow 0$.

#### Proof of Proposition 1

First note that the response curves are continuous functions of the parameters. This fact implies that the possible limiting curves that are not branches of hyperbola on $\mathcal {X}$ may only be found for the parameters that tend to the border of their domain. Clearly, these curves will be monotone nondecreasing.

$$\tilde{\theta }_{0}$$:
for $\tilde{\theta }_{0}$ that diverges, no bounded curves are possible.
$$\tilde{\theta }_{1}$$:
for $\tilde{\theta }_{1} \rightarrow \infty$, the class of admissible bounded increasing limiting curves arises when $\tilde{\theta }_{1} = o(\tilde{\theta }_{2})$ [limit class 2] or when $\tilde{\theta }_{1} = O(\tilde{\theta }_{2})$ [limit class 1]. When $\tilde{\theta }_{1} \rightarrow 0$ and $\tilde{\theta }_{2}$ bounded away from 0, the model is not identifiable as $\tilde{\theta }_{2}$ is meaningless [limit class 2].
$$\tilde{\theta }_{2}$$:
for $\tilde{\theta }_{2} \rightarrow \infty$ and $\tilde{\theta }_{1}$ bounded from above, the model is not identifiable as $\tilde{\theta }_{1}$ is meaningless (limit class 2). For $\tilde{\theta }_{2} \rightarrow 0$, the class of admissible bounded increasing limiting curves arises when $\tilde{\theta }_{1} = o(\tilde{\theta }_{2})$ [limit class 2] and when $\tilde{\theta }_{1} = O(\tilde{\theta }_{2})$ [limit class 3].

$$\square $$

#### Remark 1

Let us note that the three limit classes in Proposition 1 correspond to common models in practice. In class 1, the mean response depends linearly on the dose; in class 2 the mean response is independent of the dose; and class 3 corresponds to the typical model for studying homeopathic therapies (where there is a dose–effect only after a null threshold $\tilde{x}=0$ or $x=a$).

## Maximum likelihood estimator

**Fig. 2:**
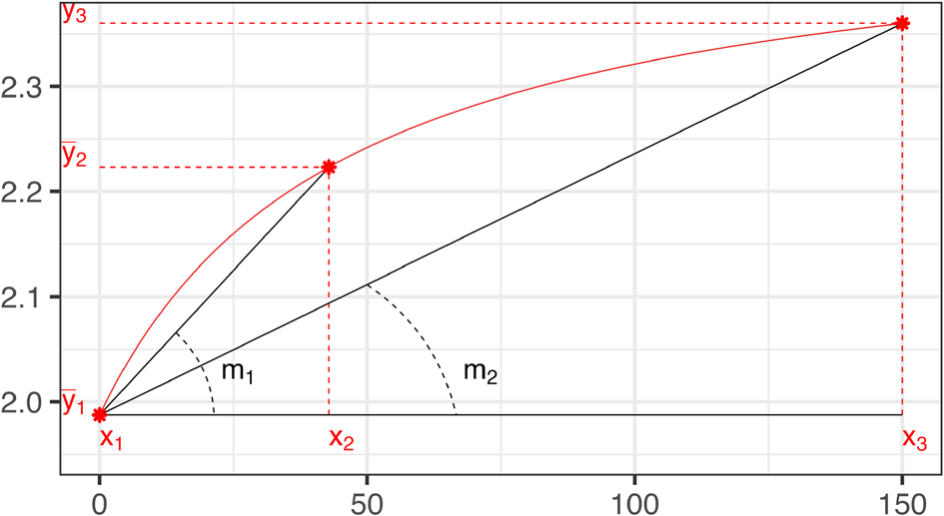
Example of points $\{(x_i,\overline{y}_{i}), i= 1,2,3\}$ that have an increasing concave shape. The red curve is the Emax mean response that fits them. (Color figure online)

In general, for a design (2) with *M* support points, the MLE can be found from the sufficient statistics $\overline{y}_{i} = (y_{i,1}+\cdots +y_{i,n_i})/n_i$ (the mean observed response value for each design point $x_i$, $i=1, \ldots M$):

7 $$\begin{aligned} \widehat{\varvec{\theta }}_{ML} =\arg \min _{\varvec{\theta }} \sum _{i=1}^M n_i [\overline{y}_{i} - \eta (x_i,\varvec{\theta })]^2 \end{aligned}$$

(see Flournoy et al. 2021). Figure 2 shows the Emax mean response curve evaluated at the MLE given data $\{(x_i,\overline{y}_{i}), i= 1,2,3\}$ that lie on a concave increasing curve.

**Fig. 3:**
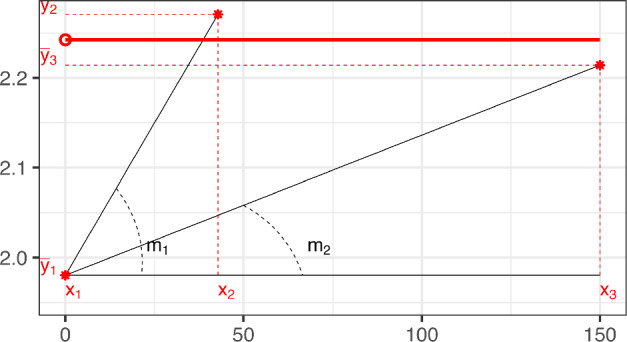
An example of three points $\{(x_i,\overline{y}_{i}), i= 1,2,3\}$ that are concave, but non-increasing, are plotted as red asterisks. An example of the family of discontinuous curve comprising case 3 of Proposition 1, which satisfies (8), is graphed in red. (Color figure online)

The next lemma states an important geometric result: an Emax model cannot be identified with a MLE when the best response fitting curve is given by a limit in Proposition 1. However, concave data sets like the one shown in Fig. 3 may occur.

### Lemma 2

If there exists a function $\eta$ in a limit class of Proposition 1 that fits the data better than any Emax model, i.e.,

8 $$\begin{aligned} \text {if for any }\varvec{\theta }\quad \sum _{i=1}^M n_i [\overline{y}_{i} - \eta (x_i)]^2 < \sum _{i=1}^M n_i [\overline{y}_{i} - \eta (x_i,\varvec{\theta })]^2, \end{aligned}$$

then the MLE does not exist.

### Proof of Lemma 2

First note that $\eta$ in a limit class of Proposition 1 means that for any $x\in \mathcal {X}$, $\eta (x) = \lim _{n\rightarrow \infty } \eta (x,\varvec{\theta }_n)$ for some sequence $\{\varvec{\theta }_n \in \Theta \}$. Then, for any $\epsilon > 0$, there exists $\varvec{\theta }$ such that

$$\begin{aligned} 0 < \sum _{i=1}^M n_i [\overline{y}_{i} - \eta (x_i)]^2 - \sum _{i=1}^M n_i [\overline{y}_{i} - \eta (x_i,\varvec{\theta })]^2 \le \epsilon ; \end{aligned}$$

and hence the minimum of (7) cannot be reached. $\square$

The next proposition proves that the probability of the non-existence of an MLE for an Emax model is always positive, regardless of the design, as the probability of (8) is non-zero in any Emax model.

### Proposition 3

For any design points $x_1<x_2<x_3<\cdots <x_M,$ the probability that the MLE does not exist is positive.

### Proof of Proposition 3

Given the design points $x_1<x_2<x_3<\cdots <x_M$, it may happen with a positive probability that the $\overline{y}_{1} \ge \overline{y}_{2} \ge \overline{y}_{3} \ge \cdots \ge \overline{y}_{M}$ since observations have Gaussian distributions.

For any fixed $\varvec{\theta }$, let $z_i= \eta (x_i,\varvec{\theta })$. Note that $z_1<z_2<z_3<\cdots <z_M$ so that $(\overline{y}_{1} , \overline{y}_{2},\ldots ,\overline{y}_{M})$ and $(z_1,z_2,\ldots ,z_M)$ are negatively correlated. Apply Lemma 12 with $y_i = \overline{y}_{i}$ to find that

$$\begin{aligned} \sum _{i=1}^M n_i [\overline{y}_{i} - \eta (x_i,\varvec{\theta })]^2> &  \sum _{i=1}^M n_i [\overline{y}_{i} - \overline{\overline{\eta }}(\varvec{\theta })]^2 \nonumber \\\ge &  \min _c \sum _{i=1}^M n_i [\overline{y}_{i} - c]^2 = \sum _{i=1}^M n_i [\overline{y}_{i} - \overline{\overline{y}}]^2, \end{aligned}$$

where

$$\begin{aligned} \overline{\overline{\eta }}(\varvec{\theta }) = \sum _{i=1}^M \left( \frac{n_i}{\sum _{j=1}^M n_j}\right) \eta ({x}_{i},\varvec{\theta }), \quad \overline{\overline{y}} = \sum _{i=1}^M \left( \frac{n_i}{\sum _{j=1}^M n_j}\right) \overline{y}_{i}. \end{aligned}$$

This means that (8) is satisfied for any M-point design with positive probability. $\square$

Clearly, the probability of the non-existent MLEs for an Emax model approaches zero as the sample size increases and/or the variance of the Gaussian noise diminishes. Classifying the geometrical situations where (8) holds is not a trivial task. In this paper, it is solved for a 3-point design, which can serve as a benchmark and a guide for other designs. For designs with more than three points, the situation becomes more complex and is left for future research. The key issue is that, given a design with two consecutive internal points, $x_*$ and $x^*$ with $n_*$ and $n^*$ experimental units, it is possible to construct a new design by merging these two points into one with $n_*+n^*$ experimental units. Which design performs better in terms of the probability of (8)? The answer is not straightforward due to the trade-off between exploring possible shapes by using more points and the uncertainty caused by reducing the number of experimental units per point.

From this point onward, we focus on the 3-point design

$$\begin{aligned} \xi =\left\{ \begin{matrix} x_1 & x_2 & x_3 \\ \omega _1 & \omega _2 & \omega _3 \end{matrix} \right\} , \end{aligned}$$

where $x_1=a$ and $x_3=b$. We characterize the geometrical situations in which the MLE cannot be computed. Note that the D-optimal design (3), in particular, is a equally supported 3-point design, where $x_2=x^*(\theta _2)$ given by Eq. (4). The following remark highlights the special role of $x_2=x^*(\theta _2)$ for the model identification.

### Remark 2

**Fig. 4:**
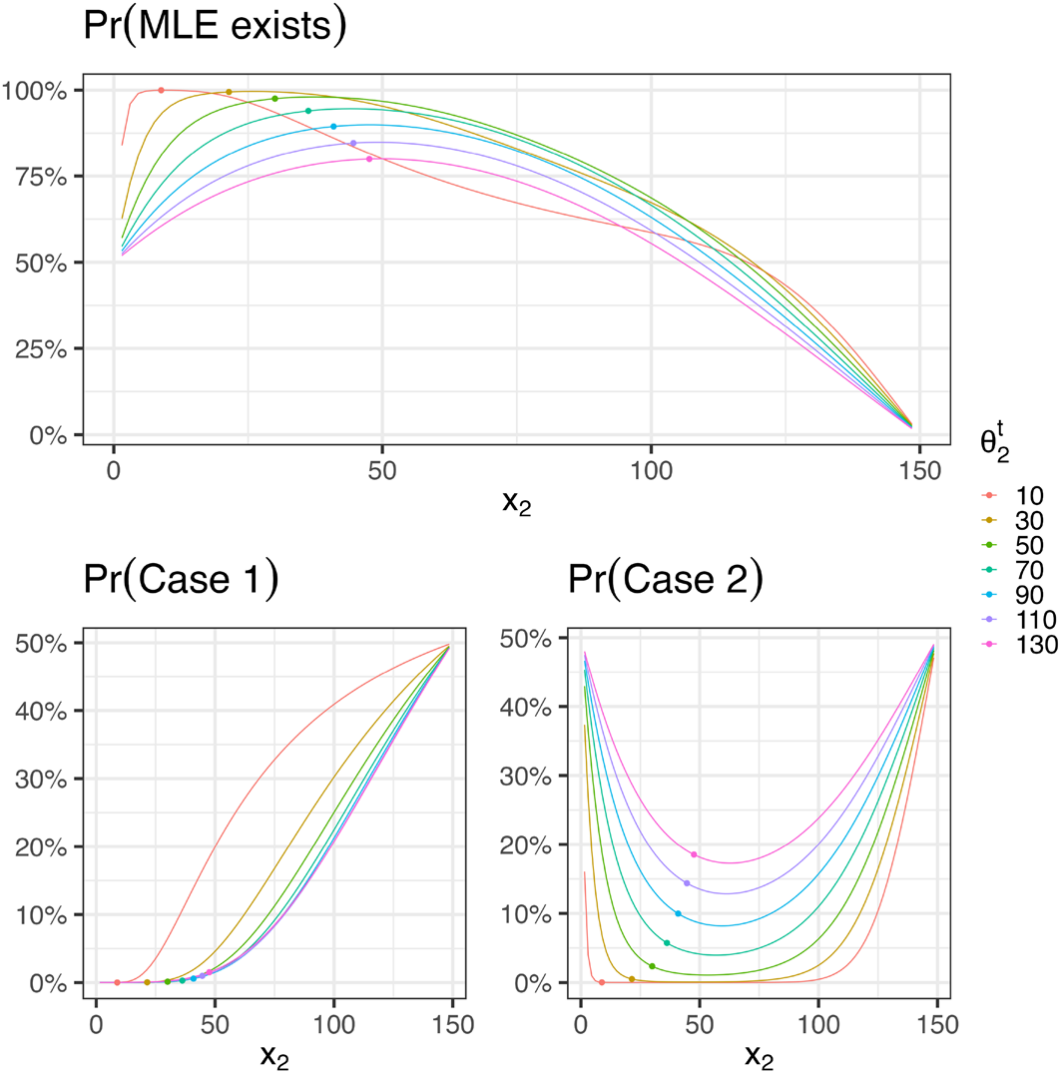
Probability that the MLE exists (top figure, see Remark 3) and that the MLE does not due to Cases 1 and 2 (bottom left and right figures, respectively; see Remarks 4 and 5), as a function of the central point $x_2$ for different values of $\theta _2^t$, where $\theta _0^t=2$, $\theta _1^t=0.467$, $a=0.001$, $b=150$, $\sigma =0.1$, $n_i=6$ (with $i=1,2,3$). The dot on the curves corresponds to the D-optimal central point $x^*(\theta _2^t)$

That $x^*(\theta _2) \in (a, (a+b)/2)$ follows from Eq. (4) since $\theta _2>-a$. To show this, one can write down the target inequality

$$\begin{aligned} x^*(\theta _2) \le (a+b)/2, \end{aligned}$$

and after simple equivalent transformations of both sides of the inequality, get $(a-b)^2\ge 0$. Looking at Case 1 and Case 2 in Fig. 4, one can see that shifting $x_2$ a bit below the mid point increases the probability that the MLE exists. The fact that the two extreme points of $\xi ^*$ lie at the boundary of $\mathcal {X}$ is not surprising; but it is interesting to note that $x^*(\theta _2)$ is the point where we observe the average value of the minimum and maximum mean responses, i.e. $\eta \left( x^*(\theta _2),\varvec{\theta }\right) =[\eta (a,\varvec{\theta })+\eta (b,\varvec{\theta })]/2$. This property also increases the probability that $\overline{y}_1< \overline{y}_2 < \overline{y}_3$, and this explains why the D-optimal design (by balancing the two cases given in Fig. 4) reduces the probability that MLEs don’t exist.

In what follows, we show that if the three points $\{(x_i,\overline{y}_{i}), i= 1,2,3\}$ have an increasing concave shape (that is, the three points lay on an increasing concave curve as in Fig. 2), then there exists a unique analytic solution of the MLE. Otherwise, the existence of the MLE fails and we provide the probability of observing data in the different adverse situations (see Fig. 4).

### An analytic solution of the MLE for data with increasing concave shape

The mathematical condition for the data having a concave shape is given by $m_1 > m_2$, where

$$\begin{aligned} m_1 = \frac{\overline{y}_{2}- \overline{y}_{1} }{ x_{2} - x_{1}} \quad \text {and}\quad m_2 = \frac{\overline{y}_{3}- \overline{y}_{1} }{ x_{3} - x_{1}}; \end{aligned}$$

the condition for the data having an increasing shape is given by $\overline{y}_{1}< \overline{y}_{2} < \overline{y}_{3}$. Figure 2 displays a geometric visualization of these conditions.

#### Remark 3

The mathematical conditions for data to have an increasing and concave shape can be rewritten as a linear inequality $A\overline{\textbf{y}} < \textbf{0}$, with

$$\begin{aligned} A =\begin{pmatrix} \Big (\tfrac{1}{x_2-x_1} -\tfrac{1}{x_3-x_1}\Big ) & -\tfrac{1}{x_2-x_1} & \tfrac{1}{x_3-x_1} \\ 1 & -1& 0\\ 0 & 1& -1\end{pmatrix}, \quad \overline{\textbf{y}} = \begin{pmatrix} \overline{y}_{1} \\ \overline{y}_{2} \\ \overline{y}_{3} \end{pmatrix}. \end{aligned}$$

Since $\overline{y}_{i} \sim N( \eta (x_i,\varvec{\theta }), {\sigma ^2}/{n_i})$ are independent, we are able to compute numerically the probability of $P(A\overline{\textbf{y}} < \textbf{0})$. The top plot in Fig. 4 displays the probability that the MLE exists for various values of the central support point $x_2$.

The following results provide the analytic solution of the unique MLE for the reparametrization given in (5) and for the model parameterization in (1).

#### Theorem 4

Under the Emax model and a three points design, if the data have increasing concave shape, then the MLE of $\tilde{\varvec{\theta }}$ is given by

$$\begin{aligned} \begin{aligned} \tilde{\theta }_{ML,0}&= \overline{y}_{1},\\ \tilde{\theta }_{ML,1}&= \frac{ m_1 m_2 }{m_1-m_2} (\tilde{x}_3 -\tilde{x}_2 ),\\ \tilde{\theta }_{ML,2}&= \frac{ \overline{y}_{3} - \overline{y}_{2} }{m_1-m_2}. \end{aligned} \end{aligned}$$

#### Proof of Theorem 4

Equation (7) can be rewritten in $\tilde{\varvec{\theta }}$ and will be referenced in this parameterization during this proof. From Eq. (7), with the new coordinates $\tilde{x} = x-a$,

9a $$\begin{aligned} \overline{y}_{1}&=\tilde{\theta }_{ML,0} + \tilde{\theta }_{ML,1}\, \frac{\tilde{x}_{1}}{\tilde{x}_{1}+\tilde{\theta }_{ML,2}}, \end{aligned}$$

9b $$\begin{aligned} \overline{y}_{2}&= \tilde{\theta }_{ML,0} + \tilde{\theta }_{ML,1}\, \frac{\tilde{x}_{2}}{\tilde{x}_{2}+\tilde{\theta }_{ML,2}}, \end{aligned}$$

9c $$\begin{aligned} \overline{y}_{3}&= \tilde{\theta }_{ML,0} + \tilde{\theta }_{ML,1}\, \frac{\tilde{x}_{3}}{\tilde{x}_{3}+\tilde{\theta }_{ML,2}}. \end{aligned}$$

If the system of the three equations $\{\overline{y}_{i} = \eta (x_i,\tilde{\varvec{\theta }} ),$ $i=1,2,3\}$ is solved for a unique $\tilde{\varvec{\theta }}$, then this solution must be a MLE. Recalling that $\tilde{x}_1 = 0$, $m_1= (\overline{y}_{2}-\overline{y}_{1})/\tilde{x}_2$ and $m_2= (\overline{y}_{3}-\overline{y}_{1})/\tilde{x}_3$, then the system (9) is equivalent to 
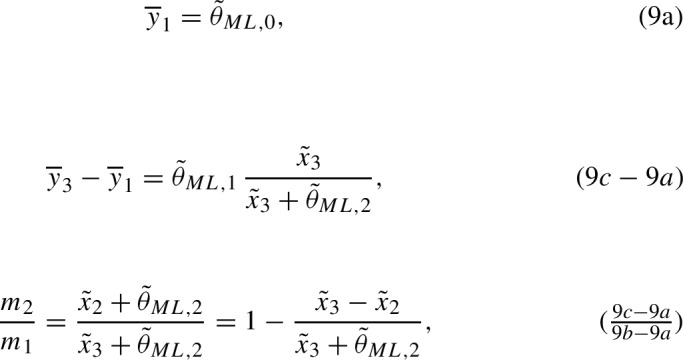
 and the thesis follows. $\square$

#### Corollary 5

Under the conditions of Theorem 4, the MLE of ${\varvec{\theta }}$ is

$$\begin{aligned} \widehat{\theta }_{ML,0}&= \overline{y}_{1} -a \frac{ m_1m_2(b-x_2)}{ (\overline{y}_{3} - \overline{y}_{2}) - a (m_1-m_2)},\\ \widehat{\theta }_{ML,1}&= \frac{ m_1 m_2 }{m_1-m_2} (b-x_2) + a \frac{ m_1m_2(b-x_2)}{ (\overline{y}_{3} - \overline{y}_{2}) - a (m_1-m_2)},\\ \widehat{\theta }_{ML,2}&= \frac{ \overline{y}_{3} - \overline{y}_{2} }{m_1-m_2} - a. \end{aligned}$$

#### Proof of Corollary 5

Express (6) in terms of $\varvec{\theta }$ and apply Theorem 4. $\square$

We have proved that, for data with increasing concave shape, the graph of $\eta (\cdot ,\widehat{\varvec{\theta }}_{ML})$ contains the three points $\{(x_i,\overline{y}_{i})$, $i = 1,2,3\}$ because of system (9). This will not be the case for other shapes; when (9) is not satisfied, we will always find a function $\eta$ in the limit class of Proposition 1 such that (8) holds for three point designs. Then the MLE will not exist by Lemma 2.

If we include the limit case in the model when there is no effect of the drug, that is, a constant response is obtained with $\theta _1=0$, then the model is not identifiable: Eq. (7) may have more than one minimum. Another limit case, which is also not identifiable, is the linear increasing model (see 2 in Proposition 1), that can be included only by a reparametrization.

### Cases for which the MLE does not exist

**Fig. 5:**
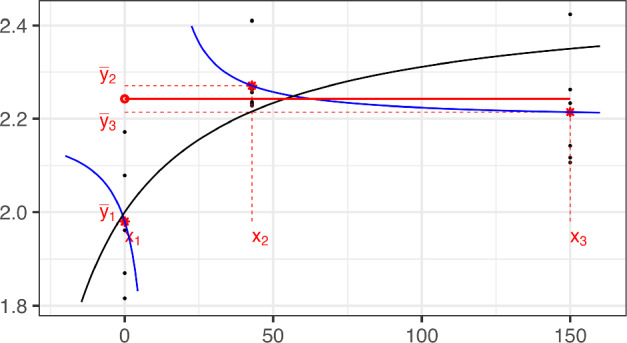
Case 1: data with non-increasing concave shape. The black line represents the true Emax model that generates the sample data (black dots). The red star points are the sample means ($\overline{y}_i$) at the experimental points $\{x_i, i=1,2,3\}$. The blue curve displays the hyperbole that fits the three points $\{(x_i,\overline{y}_i), i=1,2,3\}$ [i.e. model (1) without any parametric constraint]. The discontinuous curve given in (12), which satisfies (8), is plotted in red. (Color figure online)

Two data configurations in which the data do not exhibit an increasing concave shape lead to non-existent MLEs:

**Case 1:** the data exhibit a concave shape ($m_1>m_2$), but the three means $\{\overline{y}_{i}$, $i = 1,2,3\}$ are not increasing (see Fig. 5);

#### Remark 4

The mathematical conditions for a non-increasing concave shape with $\overline{y}_{1} < \overline{y}_{23}$) (first subcase in Proposition 6) can be rewritten as a linear inequality $A\overline{\textbf{y}} < \textbf{0}$, with

$$\begin{aligned} A =\begin{pmatrix} \tfrac{1}{x_2-x_1} -\tfrac{1}{x_3-x_1} & -\tfrac{1}{x_2-x_1} & \tfrac{1}{x_3-x_1} \\ 1 & -\frac{n_2}{n_2+n_3} & -\frac{n_3}{n_2+n_3} \\ 0 & -1 & 1 \end{pmatrix}, \quad \overline{\textbf{y}} = \begin{pmatrix} \overline{y}_{1} \\ \overline{y}_{2} \\ \overline{y}_{3} \end{pmatrix}, \end{aligned}$$

while the mathematical condition of non-increasing concave shape with $\overline{y}_{1} \ge \overline{y}_{23}$ (second subcase in Proposition 6) can be rewritten as $A\overline{\textbf{y}} < \textbf{0}$, with

$$\begin{aligned} A =\begin{pmatrix} \tfrac{1}{x_2-x_1} -\tfrac{1}{x_3-x_1} & -\tfrac{1}{x_2-x_1} & \tfrac{1}{x_3-x_1} \\ -1 & +\frac{n_2}{n_2+n_3} & +\frac{n_3}{n_2+n_3} \\ 0 & -1 & 1 \end{pmatrix} , \quad \overline{\textbf{y}} = \begin{pmatrix} \overline{y}_{1} \\ \overline{y}_{2} \\ \overline{y}_{3} \end{pmatrix}. \end{aligned}$$

In both subcases, $\overline{y}_{i} \sim N( \eta (x_i,\varvec{\theta }), {\sigma ^2}/{n_i})$ are independent and the probabilities that observed data will belong to each subcase can be computed. The bottom left plot in Fig. 4 displays the probability that the MLE fails (as a function of the central support point $x_2$) due to non-increasing concave data.

**Fig. 6:**
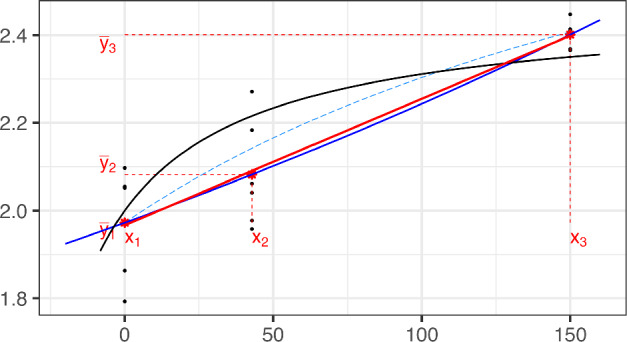
Case 2: data with convex shape. The black line represents the true Emax model that generates the sample data (black dots). The red star points are the sample means ($\overline{y}_i$) at the experimental points $\{x_i, i=1,2,3\}$. The blue curve displays the hyperbole that fits the three points $\{(x_i,\overline{y}_i), i=1,2,3\}$ [i.e. model (1) without any parametric constraint]. The simple linear regression of the original data, which satisfies (8), is plotted in red. The light blue dashed-curve represents the Emax model that corresponds to the Firth-modified estimator. (Color figure online)

**Case 2:** the three observed means have convex shape ($m_1\le m_2$); see Fig. 6.

#### Remark 5

The mathematical condition of data with convex shape (Case 2) can be rewritten as a linear inequality $A\overline{\textbf{y}} < \textbf{0}$ with

$$\begin{aligned} P (m_1 \le m_2) = P \Big ( \big (\tfrac{1}{x_2-x_1} -\tfrac{1}{x_3-x_1}\big )\overline{y}_{1} -\tfrac{1}{x_2-x_1} \overline{y}_{2} + \tfrac{1}{x_3-x_1} \overline{y}_{3} \ge 0 \Big ), \end{aligned}$$

where $\overline{y}_{i} \sim N( \eta (x_i,\varvec{\theta }), \sigma ^2/n_i)$ are independent. The bottom right plot in Fig. 4 displays the probability that the MLE fails (as a function of the central design point $x_2$) due to convex data.

**Case 1: Data with non-increasing concave shape.** In this case $m_1>m_2$, but the MLE existence requirement that $\overline{y}_{1}< \overline{y}_{2} < \overline{y}_{3}$ fails.

We start by showing that, in this case, $\overline{y}_{2} \ge \overline{y}_{3}$. In fact, by contradiction, an assumption that $\overline{y}_{2} < \overline{y}_{3}$ implies $m_2>0$. Hence $m_1>0$ which implies that $\overline{y}_{1} < \overline{y}_{2}$, which together with the assumption $\overline{y}_{2} < \overline{y}_{3}$ leads to the contradiction.

The fact is that $\overline{y}_{2} \ge \overline{y}_{3}$ is opposite to the expected response from the Emax model, where $\eta ({x}_2,{\varvec{\theta }}) < \eta ({x}_3,{\varvec{\theta }})$. Applying Corollary 13 with $y_1=\overline{y}_2$, $y_2=\overline{y}_3$, $z_1=\eta ({x}_2,{\varvec{\theta }})$, $z_2=\eta ({x}_3,{\varvec{\theta }})$, $N_1=n_2$ and $N_2=n_3$ we obtain

11 $$\begin{aligned} &  n_2 \left[ \overline{y}_{2} - \eta ({x}_2,{\varvec{\theta }})\right] ^2 + n_3 \left[ \overline{y}_{3} - \eta ({x}_3,{\varvec{\theta }})\right] ^2 > n_2 (\overline{y}_{2} - c)^2 + n_3 (\overline{y}_{3} - c)^2\nonumber \\ &  \quad \ge \min _d \sum _{i=2,3} n_i (\overline{y}_{i} - d )^2 = \sum _{i=2,3} n_i \big (\overline{y}_{i} - \overline{y}_{23} \big )^2, \end{aligned}$$

where

$$\begin{aligned} \overline{y}_{23}=\frac{n_2\overline{y}_{2} + n_3\overline{y}_{3}}{n_2+n_3}. \end{aligned}$$

Equation (11) states that any curve from the Emax model fits the two points $({x}_2,\overline{y}_{2})$ and $({x}_3,\overline{y}_{3})$ worse than the horizontal line $y = \overline{y}_{23}$. To complete (7), it remains to include in (11) the term that relates to $(x_1,\overline{y}_{1})$. To do so, we consider two subcases that are distinguished by the position of $\overline{y}_{1}$ with respect to $\overline{y}_{23}$. By Lemma 2, the MLE does not exist in either subcase.

#### Proposition 6

Subcase $\overline{y}_{1} < \overline{y}_{23}$: when $\overline{y}_{1} < \overline{y}_{23},$ the function

12 $$\begin{aligned} \eta (x) ={\left\{ \begin{array}{ll} \overline{y}_{1} & \text {if}\, x = x_1=a;\\ \overline{y}_{23} & \text {if}\, x \in (x_1,b]; \end{array}\right. } \end{aligned}$$

satisfies (8) and $\eta$ belongs to the limit class 3 of Proposition 1.

Subcase $\overline{y}_{1} \ge \overline{y}_{23}$: When $\overline{y}_{1} \ge \overline{y}_{23},$ the horizontal line

13 $$\begin{aligned} y = \overline{y}, \quad \textrm{where}\; \overline{y}=\frac{n_1\overline{y}_{1} + n_2\overline{y}_{2} + n_3\overline{y}_{3}}{n_1+n_2+n_3}, \end{aligned}$$

satisfies (8) and $\eta$ belongs to the limit class 2 of Proposition 1.

#### Proof of Proposition 6

Subcase $\overline{y}_{1} < \overline{y}_{23}$. In this case, for any $\varvec{\theta }$, by (11) we obtain

$$\begin{aligned} &  \sum _{i=1}^3 n_i [\overline{y}_{i} - \eta (x_i,\varvec{\theta })]^2> \sum _{i=2,3} n_i [\overline{y}_{i} - \eta (x_i,\varvec{\theta })]^2\\ &  \quad > \sum _{i=2,3} n_i [\overline{y}_{i} - \overline{y}_{23}]^2 = \sum _{i=1}^3 n_i [\overline{y}_{i} - \eta (x_i)]^2 , \end{aligned}$$

where $\eta$ is defined in Eq. (12).

Subcase $\overline{y}_{1} \ge \overline{y}_{23}$. Equation (A3) with $y_1=\overline{y}_2$, $y_2=\overline{y}_3$, $z_1=\eta ({x}_2,{\varvec{\theta }})$, $z_2=\eta ({x}_3,{\varvec{\theta }})$, $N_1=n_2$ and $N_2=n_3$ gives

14 $$\begin{aligned} &  \sum _{i=1,2,3} n_i [\overline{y}_{i} - \eta (x_i,\varvec{\theta })]^2 = n_1 [\overline{y}_{1} - \eta (x_1,\varvec{\theta })]^2 + \sum _{i=2,3} n_i [\overline{y}_{i} - \eta (x_i,\varvec{\theta })]^2\nonumber \\ &  \quad > n_1 [\overline{y}_{1} - \eta (x_1,\varvec{\theta })]^2 + \sum _{i=2,3} n_i \Big (\overline{y}_{i} - \frac{n_2\eta (x_2,\varvec{\theta }) + n_3\eta (x_3,\varvec{\theta })}{n_2+n_3} \Big )^2. \end{aligned}$$

Let $\overline{\eta }_{23}(\varvec{\theta }) = [n_2\eta (x_2,\varvec{\theta }) + n_3\eta (x_3,\varvec{\theta })]/(n_2+n_3)$. The monotonicity of Emax model for $\theta _1>0$ implies that $\eta (x_1,\varvec{\theta }) < \overline{\eta }_{23}(\varvec{\theta })$. We have to prove that, for any $\varvec{\theta }$, there exists a constant $c = c(\theta )$ such that

15 $$\begin{aligned} \sum _{i=1,2,3} n_i [\overline{y}_{i} - \eta (x_i,\varvec{\theta })]^2\ > \sum _{i=1,2,3} n_i [\overline{y}_{i} - c(\theta )]^2. \end{aligned}$$

We consider two subcases separately:

*Subcase*$$\overline{\eta }_{23}(\varvec{\theta }) \le \overline{y}_{1}$$:
In this case $\eta (x_1,\varvec{\theta }) < \overline{\eta }_{23}(\varvec{\theta }) \le \overline{y}_{1}$. So $[\overline{y}_{1} - \eta (x_1,\varvec{\theta })]^2 > [\overline{y}_{1} - \overline{\eta }_{23}(\varvec{\theta })]^2$. By Eq. (14), we obtain
  $$\begin{aligned} \sum _{i=1,2,3} n_i [\overline{y}_{i} - \eta (x_i,\varvec{\theta })]^2\ > \sum _{i=1,2,3} n_i \Big (\overline{y}_{i} - \overline{\eta }_{23}(\varvec{\theta }) \Big )^2. \end{aligned}$$
$$Subcase\ \overline{\eta }_{23}(\varvec{\theta }) > \overline{y}_{1}$$:
In this case, $\overline{\eta }_{23}(\varvec{\theta }) > \overline{y}_{1} \ge \overline{y}_{23}$. Since $\small {f(c) = \sum _{i=2,3}}$ $\small {n_i (\overline{y}_{i} - c )^2}$ is a parabola with minimum at $\overline{y}_{23}$, then $f(\overline{\eta }_{23}(\varvec{\theta })) > f(\overline{y}_{1})$. Hence by (14)
  $$\begin{aligned} \sum _{i=1,2,3} n_i [\overline{y}_{i} - \eta (x_i,\varvec{\theta })]^2\ > \ [\overline{y}_{1} - \eta (x_1,\varvec{\theta })]^2 + f(\overline{y}_{1})\ \ge \sum _{i=1,2,3} n_i (\overline{y}_{i} - \overline{y}_{1} )^2. \end{aligned}$$

By (15),

$$\begin{aligned} \sum _{i=1,2,3} n_i [\overline{y}_{i} - \eta (x_i,\varvec{\theta })]^2\ > \ \min _c \sum _{i=1,2,3} n_i (\overline{y}_{i} - c)^2 \ = \ \sum _{i=1,2,3} n_i (\overline{y}_{i} - \overline{y})^2 , \end{aligned}$$

where $\overline{y}$ is the weighted mean of $\{\overline{y}_{i}$, $i=1,2,3\}$ and thus, Eq. (13) holds. $\square$

**Fig. 7:**
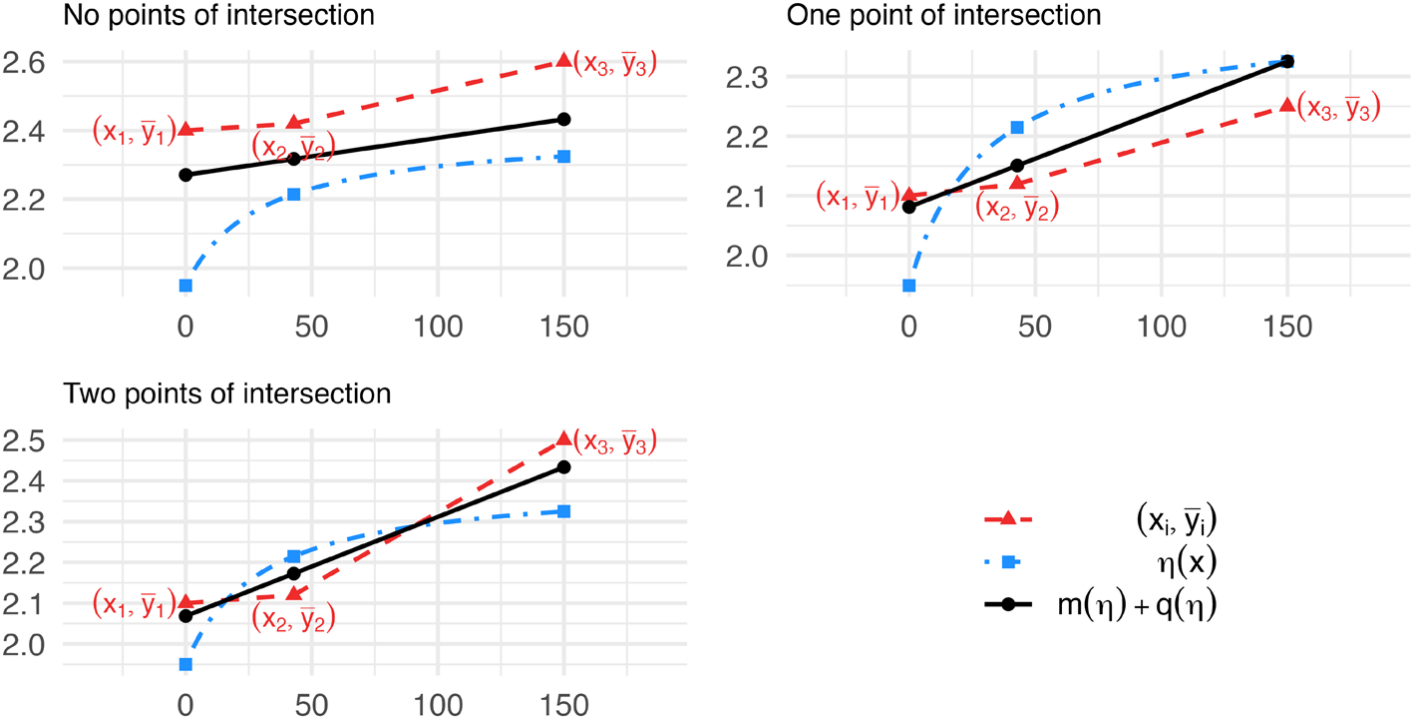
Examples of Case 2 non-existent MLEs providing the concept of the proof of Lemma 9: the red triangles represent the data $\{(x_i,\overline{y}_i)$, $i=1,2,3\}$ that exhibit a convex shape. $\eta$ (blue dash-dotted line) is a concave increasing curve that might fit the data. The black straight line fits the three red-triangles better than $\eta$ (see Lemma 9). (Color figure online)

**Case 2: Data with convex shape.** Data with a convex shape ($m_1 \le m_2$) is not expected from the Emax model whose response curve is concave. This fact implies that any curve $\eta (x,\varvec{\theta })$ from the Emax model fits the three points $\{({x}_i,\overline{y}_{i}), i=1,2,3\}$ worse than a specific nondecreasing line, as is proved in Lemma 9 in Sect. A of Auxiliary results. We display the concept of Lemma 9 in Fig. 7. The results for two subcases are described below.

Let $\eta _0(x) = m_0 x+ q_0$ be the *ordinary weighted simple linear regressor* of $(x_i,\overline{y}_i)$ $i=1,2,3$ – that is the *simple linear regressor* of the original data.

*Subcase*$$m_0>0$$:
Equation (8) is satisfied with $\eta _0(x)$ since, by Lemma 9,
  $$\begin{aligned} \sum _{i=1,2,3} n_i [\overline{y}_{i} - \eta (x_i,\varvec{\theta })]^2\> &  \sum _{i=1,2,3} n_i \big \{\overline{y}_{i} - [m(\eta )x_{i} + q(\eta )] \big \}^2 \\\ge &  \sum _{i=1,2,3} n_i \big [\overline{y}_{i} - \eta _0(x_i)\big ]^2 . \end{aligned}$$
  Moreover, $\eta _0$ belongs to limit class 1 of Proposition 1.
*Subcase*$$m_0\le 0$$:
Note that all the lines in (A1) have $m(\eta )\ge 0$ by Lemma 9, since $\eta (x,\varvec{\theta })$ is increasing, and hence the left derivative of $\eta (x,\varvec{\theta })$ in $x_3$ is positive. Then, by Corollary 11, for any $\varvec{\theta }$ there exists a constant $c(\varvec{\theta })$ such that
  $$\begin{aligned} \sum _{i=1,2,3} n_i \left\{ \overline{y}_{i} - [m(\eta )x_{i} + q(\eta )] \right\} ^2 \ge \ \sum _{i=1,2,3} n_i \left[ \overline{y}_{i} - c(\varvec{\theta }) \right] ^2 , \end{aligned}$$
  so that, by Lemma 9,
  $$\begin{aligned} \sum _{i=1,2,3} n_i [\overline{y}_{i} - \eta (x_i,\varvec{\theta })]^2 \ > \ \min _c \sum _{i=1,2,3} n_i (\overline{y}_{i} - c)^2 \ = \sum _{i=1,2,3} n_i (\overline{y}_{i} - \overline{y})^2 . \end{aligned}$$
  Equation (8) holds with $y = \overline{y}$ which belongs to the limit class 2 of Proposition 1.

For both subcases, by Lemma 2, the MLE does not exist.

## Score modification according to Firth’s theory

To overcome the unlucky cases described in Sect. 3.2, we provide Firth’s modification of the score function for the Emax model. It is known that the solution of Firth’s modified score sometimes provides a finite estimate when MLE fails (see, for instance, Kosmidis and Firth 2009a, b). In developing explicit applications, both Firth and Kosmidis have focused on categorical response models (e.g., Kosmidis and Firth 2011, 2021; Kosmidis 2017; Kosmidis et al. 2020, 2021). For exponential families in canonical parameterization, Firth (1993) showed that his modified score equations are equivalent to maximizing the likelihood penalized with Jefferys invariant prior (Jefferys 1946).

### The score vector and Fisher information

In a regression model with normal errors $\varepsilon _i=y_i-\eta (x_i,\varvec{\theta }),i=1,\ldots ,n$, the score vector $\textbf{U}=\nabla \ln \mathcal {L}_n(\varvec{\theta }; y,x)$ is

$$\begin{aligned} \textbf{U}=\dfrac{1}{\sigma ^2}\sum _{i=1}^n [y_i-\eta (x_i,\varvec{\theta })] \, \nabla \eta (x_i,\varvec{\theta }), \end{aligned}$$

where for the Emax model (1) (as shown by Dette et al. 2010)

$$\begin{aligned}&\nabla \eta (x_i,\varvec{\theta })=\left( 1,\; \dfrac{x_i}{x_i+\theta _2},\; \dfrac{-\theta _1x_i}{(x_i+\theta _2)^2} \right) ^T. \end{aligned}$$

Now suppose the *t*th component $U_t$ of the score vector $\textbf{U}$ is adjusted to

16 $$\begin{aligned} U_t^*=U_t+A_t, \quad t=1,\ldots ,p, \end{aligned}$$

where the modification is

17 $$\begin{aligned} A_t= &  \dfrac{1}{2}{{\,\textrm{trace}\,}}\{I^{-1}(P_t+Q_t)\} \quad \text {with} \end{aligned}$$

18 $$\begin{aligned} P_t= &  E(\textbf{U}\textbf{U}^TU_t) \quad \text{ and } \quad Q_t=E(-O\,U_t). \end{aligned}$$

#### Lemma 7

In a non-linear model with normal errors,

$$\begin{aligned} A_t=\dfrac{1}{2}{{\,\textrm{trace}\,}}(I^{-1}Q_t). \end{aligned}$$

#### Proof of Lemma 7

In a non-linear model with normal errors, $P_t=E(\textbf{U}\textbf{U}^TU_t)= 0$, so the result follows directly from (17). $\square$

Let *O* and *I* denote the observed and expected (Fisher) information matrices on the *p*-dimensional vector $\varvec{\theta }$, respectively. More specifically, the *ij*th element of the observed matrix information *O* is $-{\partial ^2}\ln \mathcal {L}_n(\varvec{\theta }; y,x)/{\partial \theta _i \partial \theta _j}$, which for the Emax model (1) is

$$\begin{aligned} O=\dfrac{1}{\sigma ^2}\sum _{i=1}^n \left( \begin{array}{ccc} 1 & { \frac{x_i}{{\theta _2}+x_i}} & { \frac{-\theta _1 x_i}{\left( \theta _2+x_i\right) ^2}} \\ { \frac{x_i}{\theta _2+x_i}} & { \frac{x_i^2}{\left( \theta _2 +x_i\right) ^2}} & \frac{x_i }{\left( \theta _2+x_i\right) ^2}\left( y_i-\theta _0-2\theta _1 \frac{x_i}{\theta _2+x_i}\right) \\ \frac{- \theta _1 x_i}{\left( \theta _2+x_i\right) ^2} \;\; & \frac{x_i }{\left( \theta _2+x_i\right) ^2}\left( y_i-\theta _0-2 \theta _1 \frac{x_i}{\theta _2+x_i}\right) \;\;\; & \frac{3\theta _1^2 x_i^2}{\left( \theta _2+x_i\right) ^4}\! -\! \frac{ 2\theta _1 \,x_i (y_i- \theta _0)}{(\theta _2+x_i)^3} \! \end{array}\right) . \end{aligned}$$

The expected information matrix $I=E(O)$ is

$$\begin{aligned} I=\dfrac{1}{\sigma ^2} \sum _{i=1}^n \left( \begin{array}{ccc} 1 & { \dfrac{x_i}{\theta _2+x_i}} & -{\theta _1 \dfrac{x_i}{\left( \theta _2+x_i\right) ^2}} \\ { \dfrac{x_i}{\theta _2+x_i}} & { \dfrac{x_i^2}{\left( \theta _2 +x_i\right) ^2}} & \dfrac{-\theta _1x_i^2}{\left( \theta _2+x_i\right) ^3}\\ \dfrac{- \theta _1x_i}{\left( \theta _2+x_i\right) ^2} & \dfrac{-\theta _1 x_i^2}{\left( \theta _2+x_i\right) ^3} & \dfrac{\theta _1^2x_i^2}{\left( \theta _2+x_i\right) ^4} \end{array} \right) . \end{aligned}$$

### The score modification $A_t$

The following theorem applies Firth’s additive score modifications to the Emax model.

#### Theorem 8

For the Emax model, the additive score modifications $A_t,$ $t=1,2,3$ are

$$\begin{aligned} A_1&= \frac{1}{\theta _1 D} ( V_{1,1}\, M_{1,3}-\textrm{Cov}_{12}\, M_{1,2} ),\\ A_2&= \frac{1}{\theta _1 D} (V_{1,1}\,M_{2,4}-\textrm{Cov}_{12}\,M_{2,3}),\\ A_3&= -\frac{1}{D} (V_{1,1}\,M_{2,5} -\textrm{Cov}_{12}\,M_{2,4}), \end{aligned}$$

where for $l_1 = 1,2$ and $l_2=1,\ldots ,5,$

$$\begin{aligned} &  M_{l_1,l_2} = \textrm{E}_\xi \Big [ \frac{x_i^{l_1}}{(\theta _2+x_i)^{l_2}}\Big ]; \quad V_{l_1,l_2} = \textrm{Var}_\xi \Big [ \frac{x_i^{l_1}}{(\theta _2+x_i)^{l_2}}\Big ] = M_{2l_1,2l_2}-M_{l_1,l_2}^2;\quad \text { and} \\ &  \textrm{Cov}_{12} = \textrm{Cov}_\xi \Big [ \frac{x_i}{(\theta _2+x_i)}, \frac{x_i}{(\theta _2+x_i)^2} \Big ] = M_{2,3}-M_{1,1}\, M_{1,2}; \\  &  D = V_{1,1}\,V_{1,2}-\textrm{Cov}_{12}^2. \end{aligned}$$

#### Proof of Theorem 8

From Eq. (18), after some computation, we find that all items in the matrices $Q_t=\{Q_{t(i,j)}\}_{i,j=1,2,3}$, $t=1,2,3$, are null except those in positions (2, 3) and (3, 3). Specifically,

19 $$\begin{aligned} Q_{t(i,j)}=0 \quad \text{ for }\, (i,j)\ne (2,3)\, \text{ or }\, (3,3)\quad \text{ and }\quad t=1,2,3 \end{aligned}$$

while

$$\begin{aligned} Q_{1(2,3)}&= -\frac{1}{\sigma ^2}\sum _{i=1}^n \frac{x_i}{(x_i+\theta _2)^2} \!=\! -\frac{n}{\sigma ^2}\, M_{1,2};\\ Q_{1(3,3)}&= \frac{2\,\theta _1}{\sigma ^2} \sum _{i=1}^n \!\frac{x_i}{(x_i+\theta _2)^3} \!=\! \frac{2\,n\,\theta _1}{\sigma ^2}\, M_{1,3};\\ Q_{2(2,3)}&=-\frac{1}{\sigma ^2}\sum _{i=1}^n \dfrac{x_i^2}{(x_i+\theta _2)^3} = -\frac{n}{\sigma ^2} \, M_{2,3};\\ Q_{2(3,3)}&=\frac{2\,\theta _1}{\sigma ^2} \sum _{i=1}^n \dfrac{x_i^2}{(x_i+\theta _2)^4} =\frac{2\,n\,\theta _1}{\sigma ^2} \, M_{2,4}; \\ Q_{3(2,3)}&=\dfrac{\theta _1}{\sigma ^2}\sum _{i=1}^n \dfrac{x_i^2}{(x_i+\theta _2)^4} =\dfrac{n\,\theta _1}{\sigma ^2}\, M_{2,4};\\ Q_{3(3,3)}&= -\dfrac{2\,\theta _1^2}{\sigma ^2}\sum _{i=1}^n \dfrac{x_i^2}{(x_i+\theta _2)^5} =-\dfrac{2\,n\,\theta _1^2}{\sigma ^2} \, M_{2,5}. \end{aligned}$$

It follows from Lemma 7 for the Emax model, that

20 $$\begin{aligned} A_t=\dfrac{1}{2}{{\,\textrm{trace}\,}}(I^{-1}Q_t)=I^{-1}_{(2,3)}\,Q_{t(2,3)}+\dfrac{1}{2}\,I^{-1}_{(3,3)}\,Q_{t(3,3)}, \end{aligned}$$

where $I_{(i,j)}^{-1}$ denotes the (*i*, *j*)th element of the inverse of the expected information matrix $I^{-1}$ and the second equality follows from (19). The computation of $A_t$, $t=1,2,3$, requires only the elements in positions (2, 3) and (3, 3) of the inverse of the Fisher information matrix. Therefore, we have partitioned *I* as follows

21 $$\begin{aligned} I=\begin{pmatrix} \mathcal {I}_{11} & \mathcal {I}_{12}\\ \mathcal {I}_{21} & \mathcal {I}_{22} \end{pmatrix}, \end{aligned}$$

where

$$\begin{aligned} &  \mathcal {I}_{11}=\dfrac{n}{\sigma ^2}, \quad \mathcal {I}_{21}=\dfrac{1}{\sigma ^2} \begin{pmatrix} \displaystyle \sum \nolimits _{i=1}^n \dfrac{x_i}{\theta _2+x_i} \\ -\theta _1 \displaystyle \sum \nolimits _{i=1}^n \dfrac{x_i}{\left( \theta _2+x_i\right) ^2} \end{pmatrix} = \dfrac{n}{\sigma ^2} \begin{pmatrix} M_{1,1} \\ -\theta _1\, M_{1,2} \end{pmatrix}, \\  &  \mathcal {I}_{12}=\mathcal {I}_{21}^T \quad \textrm{and}\\ &  \mathcal {I}_{22}=\dfrac{1}{\sigma ^2} \begin{pmatrix} \displaystyle \sum \nolimits _{i=1}^n \dfrac{x_i^2}{\left( \theta _2 +x_i\right) ^2} & -\theta _1 \displaystyle \sum \nolimits _{i=1}^n \dfrac{x_i^2}{\left( \theta _2+x_i\right) ^3}\\ -\theta _1 \displaystyle \sum \nolimits _{i=1}^n \dfrac{x_i^2}{\left( \theta _2+x_i\right) ^3} & \theta _1^2 \displaystyle \sum \nolimits _{i=1}^n \dfrac{x_i^2}{\left( \theta _2+x_i\right) ^4} \end{pmatrix} \\ &  \qquad \quad = \dfrac{n}{\sigma ^2} \begin{pmatrix} M_{2,2} & -\theta _1 M_{2,3}\\ -\theta _1 M_{2,3} & \theta _1^2 M_{2,4} \end{pmatrix}. \end{aligned}$$

From (21), the following formula for $I^{-1}$ applies:

$$\begin{aligned} I^{-1} = \begin{pmatrix} \mathcal {I}^{11} & \mathcal {I}^{12}\\ \mathcal {I}^{21} & \mathcal {I}^{22} \end{pmatrix}, \end{aligned}$$

where $$\mathcal {I}^{12}={\mathcal {I}^{21}}^T$$;

22 $$\begin{aligned} \mathcal {I}^{11}&= \mathcal {I}_{11}^{-1}+\mathcal {I}_{11}^{-1} \mathcal {I}_{12}\, \mathcal {I}^{22} \, \mathcal {I}_{21} \mathcal {I}_{11}^{-1}; \nonumber \\ \mathcal {I}^{21}&= - \mathcal {I}^{22}\, \mathcal {I}_{21} \mathcal {I}_{11}^{-1}; \nonumber \\ \mathcal {I}^{22}&=(\mathcal {I}_{22}-\mathcal {I}_{21} \mathcal {I}_{11}^{-1} \mathcal {I}_{12})^{-1}. \end{aligned}$$

From (22), after some algebra, one obtains

$$\begin{aligned} \mathcal {I}^{22}=\begin{pmatrix} I^{-1}_{(2,2)} & I^{-1}_{(2,3)}\\ I^{-1}_{(3,2)} & I^{-1}_{(3,3)} \end{pmatrix} = \frac{\sigma ^2}{n \, \theta _1^2\, D} \begin{pmatrix} \theta _1^2\, V_{1,2} & \theta _1\, \textrm{Cov}_{12}\\ \theta _1\, \textrm{Cov}_{12} & V_{1,1} \end{pmatrix}. \end{aligned}$$

Substituting the expressions

$$\begin{aligned} I^{-1}_{(2,3)} = \frac{\sigma ^2 }{n\,\theta _1 D} \, \textrm{Cov}_{12} \quad \textrm{and} \quad I^{-1}_{(3,3)} = \frac{\sigma ^2 }{n\,\theta _1^2 D} \, V_{1,1} \end{aligned}$$

into $Q_{t(2,3)}$ and $Q_{t(3,3)}$ in (20), we obtain:

$$\begin{aligned} A_1&= \frac{1}{\theta _1 D} (V_{1,1}\, M_{1,3}-\text {Cov}_{12}\, M_{1,2}),\\ A_2&= \frac{1}{\theta _1 D} (V_{1,1}\,M_{2,4}-\text {Cov}_{12}\,M_{2,3}),\\ A_3&= -\frac{1}{D} (V_{1,1}\,M_{2,5} -\text {Cov}_{12}\,M_{2,4}). \end{aligned}$$

$$\square $$

Note that the modification $\textbf{A}=(A_1,A_2,A_3)^T$ of the score vector depends on $\theta _1$ and $\theta _2$. Furthermore, $\textbf{A}$ is a function of the design $\xi$ which enables us to explore the impact of the design choice on the modified score $\textbf{U}^*=(U_1^*,U_2^*,U_3^*)^T$.

#### Remark 6

In the Emax model, the derivative matrix of $\textbf{U}^*$ expressed in (16) is not symmetric, and hence there is no penalized likelihood corresponding to the modified score $U^*_t$.

## Firth’s modified scores and a design-based solution for non-existent MLEs

We begin by exploring, via simulations, whether the score modification proposed by Firth overcome the non-existence of the MLE (in Cases 1 and 2) by providing admissible solutions of $\textbf{U}^*=\textbf{0}$.

To simulate the data we fix $a=0.001$, $b=150$, $\theta _0^t=2$, $\theta _1^t=0.467$, $\theta _2^t=50$, and $\sigma =0.1$ as in Dette et al. (2012). We generate 6 responses at the experimental conditions $x_1=a$, $x_2=x^*(\theta _2)$ and $x_3=b$, and compute the sample means $\overline{y}_1$, $\overline{y}_2$ and $\overline{y}_3$. When these sample data do not have an increasing concave shape, the MLE cannot be computed and we try to compute the Firth’s modified estimate. We replicate the above computations $N=10,000$ times with the goal of computing the proportion of times that:

- the MLE exists;
- the data belong to Case 1 and Firth’s modification succeeds in finding an admissible estimate;
- the data belong to Case 2 and Firth’s modification succeeds in finding an admissible estimate.

To explore the dependence of these proportions on the choice of guessed values of $\theta _2$, say $\theta _2^g$, we replicate the above simulation study for $\theta _2^g\in \{12.5, 25, 50, 75, 100 \}$.

Table 1 displays the simulation results. From the second column we can immediately observe that the proportion of times that the MLE exists is consistent with the theoretical probability (described in Remark 3) which is reported in parenthesis. The same consistency is displayed in columns 3 and 5, for Case 1 and Case 2, respectively.

**Table 1:** Percent times that the MLE exists (column 2) or does not (columns 3 and 5), with the theoretical probability reported in parenthesis

Nominal	%	%	% Firth’s success	%	% Firth’s success
$\theta _2$	MLE exists	Case 1	with Case 1	Case 2	with Case 2
12.5	84.48 (84.82)	0.00 (0.00)	NA	15.52 (15.18)	100.00
25	93.69 (93.74)	0.00 (0.01)	NA	6.31 (6.25)	99.84
50	97.50 (97.53)	0.10 (0.12)	0.00	2.40 (2.35)	99.58
75	98.12 (98.01)	0.44 (0.47)	0.00	1.44 (1.53)	100.00
100	97.91 (97.77)	0.85 (0.98)	1.18	1.24 (1.25)	100.00

Columns 4 and 6 display the proportion of times that Firth’s correction succeeds in finding an admissible estimate in Cases 1 and 2, respectively

*NA* not applicable

Case 1 instead remains unsolved, as shown in column 4: there are few Case 1 problems, but almost all of them cannot be solved by Firth’s correction. In contrast, Firth’s modified score function virtually always provides admissible estimates for data in Case 2 (see column 6).

Since Case 1 is not solved by Firth’s correction, we propose an alternative that involves choosing the experimental point $x_2$ accordingly to an hypothesis test on $\theta _2$ in the next section.

### Design choice based on a hypothesis test provides modified MLE solutions for Case 1 data

We construct a hypothesis test in which data with sample response means that display a non-increasing concave shape fall into the rejection region.

**Fig. 8:**
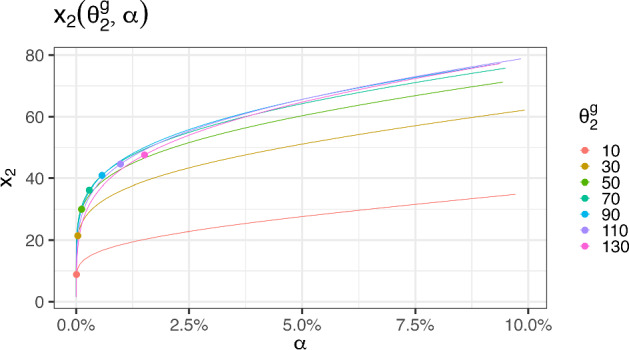
Central experimental point $x_2$ as a function of $\alpha$, for different $\theta _2^g$ (the graph has been obtained by changing *x* and *y* in Fig. 4). The dots on the curves represent the central points of the D-optimal designs $x_2^*(\theta _2^g)$ given by Eq. (4)

Consider the hypothesis test:

23 $$\begin{aligned} H_0&:\ \theta _2 \ge \theta _2^{g}, \end{aligned}$$

24 $$\begin{aligned} H_1&:\ \theta _2 < \theta _2^{g}, \end{aligned}$$

where $\theta _2^{g}$ is a guessed value such that $\theta _2^{g}>-a$ and reject the null hypothesis whenever the data belongs to Case 1. The power function of this test, $\beta (\theta _2;x_2)$, is shown in Fig. 4. It is a function of both $\theta _2$ and the experimental condition $x_2$. Therefore, given a design point $x_2$, the significance level is $\alpha =\beta (\theta _2^g;x_2)$. See Fig. 8 for a graphical representation of $x_2$ as a function of $\alpha$ and $\theta _2^g$.

Reversing the role between $x_2$ and $\alpha$, this last statement can be used by an experimenter to properly choose the value of $x_2$ that guarantees a specific significance level $\alpha$. In other words, by solving for $x_2$ the equation $\alpha =\beta (\theta _2^g;x_2)$, one obtains the central design point $x_2(\theta _2^g,\alpha )$ and by solving this equation with a small $\alpha$ guarantees a small probability of observing a bad sample belonging to Case 1.

Let us observe that the D-optimal design values for $x_2$ (the dots on the curves of Fig. 8) correspond to very low $\alpha$-values. Thus, if $H_0$ is true, fixing a low value for $\alpha$ and taking the same proportion of observations at *a*, $x_2$ and *b*, should protect from sample responses that do not produce a MLE.

If, despite choosing a small $\alpha$, we observe a bad sample belonging to Case 1, $H_1$ is more likely than $H_0$. In this last case, we suggest collecting additional data at another experimental point $x_2(\theta _2^{(1)},\alpha )\in (a,\ x_2(\theta _2^g,\alpha )), \text { for some } \theta _2^{(1)},\ \theta _2^g$ informed by Fig. 8.

## Practical guidelines and conclusions

Taking into account all the results presented in the previous sections, we can provide some simple guidelines for gathering data that produce (or are likely to produce) a finite estimate of the parameters of the Emax model.

It is well known that the D-optimal design minimizes the generalized variance of the MLE but other choices are also possible, for instance the A-optimality criterion minimizes the total variation of the MLE. Herein, we suggest using a locally D-optimal design, that is equally supported at three points, $x_1=a$, $x_2=x^*(\theta _2^{g})\in \big (a,(a+b)/2\big )$ (see Remark 2) and $x_3=b$, where $\theta _2^{g}$ is a guessed value for $\theta _2$, because it guarantees (with a large probability) the existence of the MLE, as shown in Fig. 4.

Once the responses $y_{i1},\ldots ,y_{in/3}$ (for $i=1,2,3$) have been observed at $x_1$, $x_2$ and $x_3$, respectively, we suggest to proceed as follows:

- if the sample means $\overline{y}_i$ (for $i=1,2,3$) display an increasing concave shape, then the MLE can be computed (see Theorem 4);
- if the sample means exhibit a convex shape, one should compute the Firth score modification that most likely leads to a finite parameter estimation (see Table 1);
- if the sample means exhibit a non-increasing concave shape, then it is likely that $\theta _2^g$ has been chosen too high, therefore, no estimation is expected from Firth correction (see Table 1). In this case, additional observations should be recorded at a new experimental point $x^*\big (\theta _2^{(1)}\big ) \in \big (a,x^*(\theta _2^{g})\big )$, for some value $\theta _2^{(1)}<\theta _2^{g}$, as we have the statistical evidence that $\theta _2^t$ is significantly smaller than $\theta _2^{g}$.

We believe that the theoretical contributions presented in this paper not only offer deep insights into the properties of the Emax model, but can also enhance significantly its estimation process in practical applications. Moreover, we have laid out two approaches for dealing with non-existent MLEs that can be pursued for other models.

## Appendix A Auxiliary results

In this section, we prove some additional technical results to make the paper self-contained.

### Lemma 9

Let $(x_i,\overline{y}_{i}), i=1,2,3$ be such that $x_1<x_2<x_3$ and assume

$$\begin{aligned} m_1 = \frac{\overline{y}_{2}-\overline{y}_{1}}{x_2-x_1}\ \le \ \frac{\overline{y}_{3}-\overline{y}_{1}}{x_3-x_1} = m_2. \end{aligned}$$

For any concave function $\eta :\mathcal {X}\rightarrow \mathbb {R},$ there exist a line $y = m(\eta ) x + q(\eta )$ such that

A1 $$\begin{aligned} \sum _{i=1,2,3} n_i [\overline{y}_{i} - \eta (x_i)]^2\ \ge \sum _{i=1,2,3} n_i \big \{\overline{y}_{i} - [m(\eta )x_{i} + q(\eta )] \big \}^2 , \end{aligned}$$

with the equality being possible only when $\eta$ is a segment such that $\eta (x)\equiv m(\eta )x_{i} + q(\eta )$ on $\mathcal {X}$.

Moreover, $m(\eta )$ may be chosen to be not less than the left derivative of $\eta$ in $x_3$.

### Proof of Lemma 9

Let $\zeta :\mathcal {X}\rightarrow \mathbb {R}$ be the convex function whose graph is obtained with the segments that link $(x_1,\overline{y}_1)$ with $(x_2,\overline{y}_2)$ and $(x_2,\overline{y}_2)$ with $(x_3,\overline{y}_3)$, i.e.,

$$\begin{aligned} \zeta (x) = \sup \{ f:{\mathcal {X}}\rightarrow \mathbb {R} \text { such that}\, f \text {is convex and}\, f(x_i) \le \overline{y}_i, i=1,2,3\}. \end{aligned}$$

Denote with $\textrm{graph}(\eta )$ and $\textrm{graph}(\zeta )$ the graphs of $\eta$ and $\zeta$, respectively. The main idea of this proof is to find a line $y = mx+q$ that “separates” $\textrm{graph}(\eta )$ from $\textrm{graph}(\zeta )$, which means that

A2 $$\begin{aligned} \forall x \in {\mathcal {X}}, \quad \eta (x) \le mx+q \le \zeta (x) \quad \textrm{ or } \quad \zeta (x) \le mx+q \le \eta (x) . \end{aligned}$$

Condition (A2) is sufficient to state (A1). Note that, if $\textrm{graph}(\eta )$ is not a segment, then at least one of the two points $(x_1,\eta (x_1))$ or $(x_3,\eta (x_3))$ does not belong to the line $y=mx+q$, and hence (A1) is strict. We prove (A2) for different cases.

**Case when** $\eta$ **and** $\zeta$ **are separated** (see Fig. 7 top-left plot).

Assume that $\eta \le \zeta$ on $\mathcal {X}$ or, vice versa, $\zeta \le \eta$ on $\mathcal {X}$. Then the convex hulls of their graphs are also separated. The hyperplane separation theorem ensures the existence of a line $y(x) = mx+q$ that weakly separates the two closed hulls, so that, in this case (a) $\forall x \in {\mathcal {X}}$, $\eta (x) \le mx+q \le \zeta (x)$ or (b) $\forall x \in {\mathcal {X}}$, $\zeta (x) \le mx+q \le \eta (x)$. Denote by $L_{x_3}$ the left derivative of $\eta$ in $x_3$ and observe that it bounds from below any left and right derivatives of $\eta$, since $\eta$ is concave. Assume $m<L_{x_3}$. In case (a), the line $y-(mx_3+q)=L_{x_3}(x-x_3)$ also separates $\eta$ and $\zeta$ as well as; in case (b), the line $y-(mx_1+q)=L_{x_3}(x-x_1)$ does.

**Case when** $\varvec{\eta }$ **and** $\varvec{\zeta }$ **are not separated.**

When $\eta$ and $\zeta$ are not separated, there exist two values $x_*,x^*\in {\mathcal {X}}$ such that $\eta (x_*)<\zeta (x_*)$ and $\eta (x^*)>\zeta (x^*)$. Bolzano’s Theorem ensures the existence of an interior point $x_o \in (x_1,x_3)$ such that $\eta (x_o)=\zeta (x_o)$. Note that it is not possible for there to be more than two intersecting points between $\eta$ and $\zeta$, since $\textrm{graph}(\eta )$ is strictly concave and $\textrm{graph}(\zeta )$ is convex.

We will find the separating line $y=mx+q$ by connecting two points of $\eta$, and then its slope cannot be less than the left derivative of $\eta$ in $x_3$.

**Subcase:** $x_o\in (x_1,x_3)$ **is the only point shared in common by the not-separated** $\varvec{\eta }$ **and** $\varvec{\zeta }$ (see Fig. 7 top-right plot):

Depending on the reciprocal position of $\eta$ and $\zeta$ we can have (a) $\overline{y}_3<\eta (x_3)$ and $\overline{y}_1>\eta (x_1)$ or (b) $\overline{y}_1<\eta (x_1)$ and $\overline{y}_3>\eta (x_3)$. If (a) then the line $y=mx+q$ connecting $(x_o,\eta (x_o))$ and $(x_3,\eta (x_3))$ separates $\eta$ and $\zeta$. If (b) then the line $y=mx+q$ connecting $(x_o,\eta (x_o))$ and $(x_1,\eta (x_1))$ separates $\eta$ and $\zeta$.

**Subcase:** ${x_o}$ **and** ${x^o}$ **are two distinct points shared in common by the not-separated** $\eta$ **and** $\zeta$ (see Fig. 7 bottom-left plot):

The line $y=mx+q$ connecting the two common points separates the two graphs $\eta$ and $\zeta$, and the proof is concluded. $\square$

We now show a property of the sum of squares of the residual on a generic linear set of functions.

### Proposition 10

Let $(x_i,y_i),$ $i=1,\ldots ,n$ be *n* points and let $\Theta$ be a linear set of functions, which means that $a\theta _1+b\theta _2 \in \Theta$ whenever $\theta _1,\theta _2\in \Theta$. If

$$\begin{aligned} \theta _0 = \arg \min _{\theta \in \Theta } \sum _{i=1}^n[y_i-\theta (x_i)]^2, \end{aligned}$$

then for any $\theta _1\in \Theta$ and $t\in [-1,1],$

$$\begin{aligned} \sum _{i=1}^n[y_i-\theta _1(x_i)]^2 \ge \sum _{i=1}^n\Big \{y_i-\big [t\,\theta _1(x_i)+(1-t)\,\theta _0(x_i)\big ]\Big \}^2, \end{aligned}$$

and the inequality is strict if $\theta _1\ne \arg \min _{\theta \in \Theta } \sum _{i=1}^n[y_i-\theta (x_i)]^2$ and $t\in (-1,1)$.

If $\Theta$ is a convex set, then the conclusion still holds with $t\in [0,1]$ and $t\in [0,1)$ in place of $t\in [-1,1]$ and $t\in (-1,1)$.

### Proof of Proposition 10

Given $\theta _0$ and $\theta _1$, note that

$$\begin{aligned} g(t)= &  \sum _{i=1}^n \!\Big \{y_i-\big [t\,\theta _1(x_i)+(1-t)\,\theta _0(x_i)\big ]\Big \}^2\\= &  \sum _{i=1}^n\big \{ t\,[\theta _0(x_i)-\theta _1(x_i)] + y_i-\theta _0(x_i)\big \}^2 \!\\= &  t^2 \sum _{i=1}^n [\theta _0(x_i)-\theta _1(x_i)]^2 +2t\sum _{i=1}^n [\theta _0(x_i)-\theta _1(x_i)][y_i-\theta _0(x_i)]\\ &  + \sum _{i=1}^n [y_i-\theta _0(x_i)]^2 \end{aligned}$$

defines a parabola with minimum at $t=0$ since

$$\begin{aligned} g(0) = \sum _{i=1}^n[y_i-\theta _0(x_i)]^2 = \min _{\theta \in \Theta } \sum _{i=1}^n[y_i-\theta (x_i)]^2. \end{aligned}$$

Then $g(1)=g(-1)$ and $g(t)< g(1)$ for any $t\in (-1,1)$ if $g(0)\ne g(1)$. When $\Theta$ is only a convex set, *t* can be chosen in [0, 1] to guarantee that $t\theta _1+(1-t)\theta _0\in \Theta$. $\square$

### Corollary 11

Let $(x_i,y_i),$ $i=1,\ldots ,n$ be *n* points and let $y=m_0x+q_0$ be the least square linear estimator. Then for any line $y=mx+q$ with $mm_0\le 0,$ there exists a constant *c* such that

$$\begin{aligned} \sum _{i=1}^n[y_i-(mx_i+q)]^2 \ge \sum _{i=1}^n(y_i-c)^2. \end{aligned}$$

The inequality is strict if $m\ne m_0$ or $q\ne q_0$.

### Proof of Corollary 11

Apply Proposition 10 to the set of lines, with $t^* = \vert m_0\vert /(\vert m\vert +\vert m_0\vert )$ ($0/0=0$). Since $m\vert m_0\vert + m_0\vert m\vert =0$, we get

$$\begin{aligned} \sum _{i=1}^n[y_i-(mx_i+q)]^2 \ge \sum _{i=1}^n\big [y_i-(t^*q+(1-t^*)q_0)\big ]^2, \end{aligned}$$

which is the thesis with $c=t^*q+(1-t^*)q_0$. $\square$

### Lemma 12

Let $y_1,\ldots ,y_M$ and $z_1,\ldots ,z_M$ be weighted samples with weights $n_1,\ldots ,n_M,$ and denote by $\textrm{E}_{\varvec{n}},$ $\textrm{Var}_{\varvec{n}},$ $\textrm{Covar}_{\varvec{n}}$ the weighted mean, the weighted variance and the weighted covariance, respectively. If $\textrm{Var}_{\varvec{n}}[Z]-2\textrm{Covar}_{\varvec{n}}[Y,Z]>0,$ then

$$\begin{aligned} \sum _{i=1}^M n_i(y_i-z_i)^2 > \sum _{i=1}^M n_i(y_i-\textrm{E}_{\varvec{n}}[Z])^2. \end{aligned}$$

In particular, the result is true whenever $\textrm{Covar}_{\varvec{n}}[Y,Z]<0$.

### Proof of Lemma 12

Only note that

$$\begin{aligned} \frac{ \sum _{i=1}^M n_i(y_i-z_i)^2 }{ \sum _{i=1}^M n_i }&= \textrm{E}_{\varvec{n}}[(Y-Z)^2]\\&= \textrm{E}_{\varvec{n}}[ (\{Y-\textrm{E}_{\varvec{n}}[Y]\} + \{\textrm{E}_{\varvec{n}}[Y] - \textrm{E}_{\varvec{n}}[Z]\} + \{ \textrm{E}_{\varvec{n}}[Z] - Z\} )^2 ]\\&= \textrm{Var}_{\varvec{n}}[Y] + (\textrm{E}_{\varvec{n}}[Y] - \textrm{E}_{\varvec{n}}[Z])^2 + \textrm{Var}_{\varvec{n}}[Z] -2\textrm{Covar}_{\varvec{n}}[Y,Z]\\&> \textrm{Var}_{\varvec{n}}[Y] + (\textrm{E}_{\varvec{n}}[Y] - \textrm{E}_{\varvec{n}}[Z])^2\\&= \frac{ \sum _{i=1}^M n_i(y_i- \textrm{E}_{\varvec{n}}[Z])^2 }{ \sum _{i=1}^M n_i }.&\end{aligned}$$

$$\square $$

### Corollary 13

Let $N_1$ and $N_2$ be two positive numbers, and assume $y_1\ge y_2,$ $z_1<z_2$. Then

A3 $$\begin{aligned} N_1(y_1-z_1)^2+N_2(y_2-z_2)^2 > N_1\Big (y_1-\frac{N_1z_1+N_2z_2}{N_1+N_2}\Big )^2 + N_2\Big (y_2-\frac{N_1z_1+N_2z_2}{N_1+N_2}\Big )^2, \end{aligned}$$

and in particular there exists a constant *c* such that

$$\begin{aligned} N_1(y_1-z_1)^2+N_2(y_2-z_2)^2 > N_1(y_1-c)^2 + N_2(y_2-c)^2. \end{aligned}$$

### Proof of Corollary 13

Apply Lemma 12 with $M=2$ and $n_i = N_i$, noting that $\textrm{E}_{\varvec{n}}[Z] = \frac{N_1z_1+N_2z_2}{N_1+N_2}$ and that $\textrm{Covar}_{\varvec{n}}[Y,Z]<0$. $\square$
